# Do drug treatment variables predict cognitive performance in multidrug-treated opioid-dependent patients? A regression analysis study

**DOI:** 10.1186/1747-597X-7-45

**Published:** 2012-11-02

**Authors:** Pekka Rapeli, Carola Fabritius, Hely Kalska, Hannu Alho

**Affiliations:** 1 Unit for Drug Dependence. Department of Psychiatry, Helsinki University Central Hospital, Helsinki, Finland; 2 Department of Mental Health and Substance Abuse Services, National Institute for Health and Welfare (THL), Helsinki, Finland; 3 Institute of Behavioural Sciences, University of Helsinki, Helsinki, Finland; 4 Research Unit of Substance Abuse Medicine, University of Helsinki, Helsinki, Finland

**Keywords:** Opioid-dependence, Opioid agonist therapy, Pharmacotherapy, Psychotropic drugs, Neurocognitive performance, Neuropsychological testing

## Abstract

**Background:**

Cognitive deficits and multiple psychoactive drug regimens are both common in patients treated for opioid-dependence. Therefore, we examined whether the cognitive performance of patients in opioid-substitution treatment (OST) is associated with their drug treatment variables.

**Methods:**

Opioid-dependent patients (N = 104) who were treated either with buprenorphine or methadone (n = 52 in both groups) were given attention, working memory, verbal, and visual memory tests after they had been a minimum of six months in treatment. Group-wise results were analysed by analysis of variance. Predictors of cognitive performance were examined by hierarchical regression analysis.

**Results:**

Buprenorphine-treated patients performed statistically significantly better in a simple reaction time test than methadone-treated ones. No other significant differences between groups in cognitive performance were found. In each OST drug group, approximately 10% of the attention performance could be predicted by drug treatment variables. Use of benzodiazepine medication predicted about 10% of performance variance in working memory. Treatment with more than one other psychoactive drug (than opioid or BZD) and frequent substance abuse during the past month predicted about 20% of verbal memory performance.

**Conclusions:**

Although this study does not prove a causal relationship between multiple prescription drug use and poor cognitive functioning, the results are relevant for psychosocial recovery, vocational rehabilitation, and psychological treatment of OST patients. Especially for patients with BZD treatment, other treatment options should be actively sought.

## Background

Opioid abuse affects about 0.4% of the world’s population in the age range of 15–64 years [1]. Many of them are dependent on opioids and fail to complete opioid withdrawal. Standard treatment for these individuals is opioid substitution treatment (OST), also known as opioid maintenance treatment. However, opioid-dependence is often complicated with psychiatric comorbidity. In epidemiological studies, the joint lifetime prevalence of opioid dependence and non-substance-use psychiatric disorders has ranged from almost 50% up to 90%, while the current prevalence of mood, anxiety, or personality disorders is also high [2-4]. Consequently, polypharmacy with psychoactive medication is a common practice in OST [5-9]. While several studies have examined the cognitive performance differences between buprenorphine- vs. methadone-treated opioid-dependent patients [10-14], few studies have examined the possible role of other psychoactive medications on the cognitive functioning of these patients. Thus, the results of current studies may not be fully appropriate for multidrug-treated patients. However, studies concentrating on this patient group would be important because opioid-dependent patients who are treated with multiple drugs tend to have lower quality of life than those who only use an opioid agonist drug [15]. A recent study concerning cannabis-dependent individuals found that even relatively subtle cognitive deficits that were seen in test performance can be verified by those who know the affected individual well [16]. Furthermore, at least for BZDs, even prescription drug use has been shown to be associated with serious health and safety risks [17,18]. Thus, if a drug treatment variable is negatively associated with cognitive performance in OST patients this may have practical relevance.

Earlier we have reported that opioid-dependent patients treated with buprenorphine/naloxone along with BZDs do not show attention deficit as measured by reaction time tasks, but their working memory and verbal memory performance is worse than that of healthy controls, at least for the first six months in treatment [19]. Patients treated with methadone along with BZDs show deficits in attention, working memory, and verbal memory. In our later study, OST drug group membership (buprenorphine vs. methadone) correlated significantly with attention performance and improvement in the Letter-Number Sequencing. Recent high-frequency substance abuse was associated with poor performance in working memory. Although the effects of the other two highest correlates were controlled for, many other variables could not be taken into account.

Our major aim in the current study was to examine the predictive power of drug treatment variables on specific cognitive performance measures in a naturalistic sample of multidrug-treated opioid-dependent patients. There is some evidence that short-term use of high dose methadone and BZD diazepam affects negatively on simple reaction times in opioid-dependent patients, but in buprenorphine patients only high diazepam dose affects negatively on reaction times [20,21]. Therefore, we hypothesized that in buprenorphine patients, BZD treatment (use vs. non-use or dose) but not buprenorphine treatment variables (dose) would be negatively associated with attention performance; while in methadone patients both methadone and BZD treatment variables would affect negatively on attention performance as measured by reaction times. There is preliminary evidence that buprenorphine may preserve working memory performance better than methadone [22,23]. Thus, we hypothesized that having buprenorphine as the OST drug would predict good working memory performance among opioid-dependent patients. It has been shown that long-term use of BZD drugs is associated with a wide range of cognitive deficits [24]. Sedative and anticholinergic effects have been reported for various psychiatric drugs [25,26]. Consequently, patients treated with several psychoactive drugs typically perform worse in cognitive tests than patients treated with a single drug [27,28]. We therefore hypothesized that being on a BZD drug or on a high number of prescribed psychoactive drugs (other than opioid or BZD) would predict poor cognitive performance in all measures among opioid-dependent patients treated with buprenorphine or methadone.

## Methods

The study participants were volunteer opioid-dependent patients admitted for OST in the addiction clinics of the greater Helsinki or Tampere area. Further inclusion criteria were the following: aged 18–50 years, native Finnish speaker, opioid-dependence diagnosis, and minimum six months in OST with methadone, buprenorphine, or buprenorphine/naloxone. Exclusion criteria were the following: uncontrolled polysubstance abuse, acute alcohol abuse, or acute axis I psychiatric disorder (e.g. acute phase of major depression, suicidality, hypomania, mania, or psychosis), initiation of new psychoactive drugs within the past week, severe brain injury, chronic neurological disease, history of other than substance-induced psychoses, epileptic seizures, human immunodeficiency virus (HIV) infection, pregnancy, or primary cognitive deficit (estimated IQ less than 85). To ensure study eligibility, the clinical psychiatric interview SCID I was conducted for each participant, and diagnostic criteria from the Diagnostic and Statistical Manual of Mental Disorders (DSM-IV) were applied [29]. Estimation of IQ was done by the neuropsychologist and it was based on the vocabulary subtest of the Wechsler Adult Intelligence Scale – Revised (WAIS-R) [30].

Each participant was screened for substance abuse by urine sample on the day of testing and at least once in the preceding month using the Nano5 test (from Ferle Produkter AB; Helsingborg, Sweden). Participants showing signs of current intoxication or binging on any substance of abuse and those having extra psychoactive drug doses within the last 24 h were all excluded. Also, those showing a positive drug screen for non-prescribed opioids or BZDs were excluded. The study included 104 OST patients, with 29% of them coming from the longitudinal sample used in the previous studies [5,13]. The rest were new long-term treated opioid-dependent patients. As shown in Table 1, buprenorphine-treated patients were statistically significantly younger than methadone ones, otherwise there were no significant demographic differences between buprenorphine- vs. methadone-treated patients.

**Table 1:** Group demographics

	**Buprenorphine (n = 52)**	**Methadone (n = 52)**	**Group comparisons**^**a**^
Age (*M* ± *SD*)	31 ± 7	35 ± 8	BN < M, *p* = .007**
Sex, female/male	18/34	20/32	*p =*.84
	35%/65%	38%/62%	
Verbal intelligence^b^ (*M ± SD*)	100 ± 10	101 ± 10	*p =*.44
Education, patients with primary education/any secondary education	35/17	34/18	*p =*1.00
	67%/33%	65%/35%	
Substance abuse onset age, years (M ± SD)	16 ± 3	15 ± 2	*p =* .17
Patients with early onset of substance abuse^c^	15	23	*p =*.31
	29%	44%	
Duration of OST, months (*M ± SD*)	14 ± 7	17 ± 10	*p =* .08
Patients with more than 12 month in OST	30	33	*p =* .69
	57%	63%	
Number of cognitive testing			
patients with one testing	34 (65%)	40 (77%)	*p =*.28
patients with two or three testing	18 (35%)	12 (23%)	
Patients with high-frequency substance abuse in the previous month^d^	18	22	*p =*.55
	35%	42%	
Patients with positive drug screen at test	13	13	*p =* 1.00
	25%	25%	
Patients with the past month daily nicotine use	52	51%	*p =.50*
	100%	98%	

Note. *BN* = buprenorphine patients, *M =* methadone patients.

^a^Tested with *t*-test or Fisher’s Exact Test.

^b^Estimation based on the vocabulary subtest of the Wechsler Adult Intelligence Scale – Revised (WAIS-R) [30].

^c^Considered as early up to 14 years of age.

^d^Considered as high when three or more days a week. Alcohol use was taken into account if it was at least mean weekly 16 portions (12 g) for females and 24 portions for males or binge drinking occurred on any day.

**p < 0.01.

The study was approved by both the independent Ethical Committee of the Hospital District of Helsinki and Uusimaa (permission 90/2001) and the A-Clinic Foundation. The study was conducted in accordance with the 1964 Declaration of Helsinki. All participants were able to read and understand the patient information sheet, and signed the informed consent form. The participants were free to discontinue participation in the study whenever they wanted. They were paid €20 if they attended all study visits.

### Procedure

The patients were tested with cognitive measures between three to six hours after the administration of the opioid substitution drug. In the buprenorphine group, 54% of the patients were given buprenorphine/naloxone. Thus, they received a dose of naloxone in the ratio of 1:4 combined with their buprenorphine dose. When the tablet is given sublingually the absorption of naloxone is low and eliminates within the first hours [31]. It has been shown that naloxone has minimal, if any effect, on the bioavailability or pharmacokinetics of buprenorphine [32,33]. Therefore, we combined patients using either one of the buprenorphine compounds. Benzodiazepine doses of oxazepam were converted to a diazepam equivalent dose in the ratio of 3:1 [34]. Table 2 describes the psychoactive medications used by the participants in the 24-hour period before the testing. Medication doses were compared by using the Mann–Whitney *U* test. Medication frequencies were compared using the chi-square test.

**Table 2:** Medications given to patients within the last 24 h before testing in sample I

	**Buprenorphine (n = 52)**	**Methadone (n = 52)**	**Group comparison *p*-values**
Opioid agonist drug (*M* ± *SD*)	20 ± 6 mg	−	−
Buprenorphine (*M* ± *SD*)	−	113 ± 49 mg	
			
Patients using any psychoactive medication, other than opioid	42 (81%)	43 (83%)	*p =* 1.00
Patients using any BDZ drug	37 (71%)	38 (73%)	*p =* 1.00
diazepam	29 (56%)	24 (46%)	*p =* .43
oxazepam	8 (15%)	14 (27%)	*p =* .15
Benzodiazepine, diazepam equivalent dose (*M* ± *SD*)	22 ± 10 mg	21 ± 10 mg	*p* = 0.77
The number psychoactive drugs, other than opioid or BZD^a^			
(*M* ± *SD*)	1.2 ± 1.2	1.2 ± 1.2	*p =* .81
0, no. patients (%)	18 (35%)	19 (36%)	*p* = 1.00
1 no. patients (%)	19 (37%)	13 (25%)	*p* = .29
2 no. patients (%)	9 (17%)	13 (25%)	*p* = .47
3 no. patients (%)	2 (4%)	5 (10%)	*p* = .44
4 or more no. patients (%)	4 (7%)	2 (4%)	*p* = .68
Patients using psychoactive drug, other than opioid or BZD			
Any drug^b^	34 (65%)	33 (63%)	*p =* 1.00
Anticonvulsants	6 (12%)	5 (10%)	*p =* 1.00
Antidepressants	22 (42%)	13 (25%)	*p =* .096
Antihistamines	6 (12%)	10 (19%)	*p* = .42
Neuroleptics	7 (13%)	9 (17%)	*p* = .79
Non-Benzodiazepine hypnotics	16 (31%)	19 (37%)	*p* = .68
Non-opioid pain killers	4 (8%)	6 (12%)	*p* = .74

^a^Tested with *t*-test or Fisher’s Exact Test.

^b^These included anticonvulsants (used as mood stabilizers), antidepressants, neuroleptics (used with anxiolytic indications), non-benzodiazepine hypnotics, and non-opioid pain killers.

### Statistical analyses

Group-wise comparisons of cognitive performance between buprenorphine and methadone patients were done by an analysis of variance (ANOVA). As our verbal and visual memory tests lacked age-corrected norm values and there was a significant difference between the patient groups on age, an analysis of covariance (ANCOVA) was used when testing these parameters. In all group-wise comparisons, the normality assumptions of the cognitive variables were first examined by Shapiro-Wilk’s test and the homogeneity of variance by the Levene’s test. When appropriate, analyses of the main effects were interpreted using the Welch correction for heterogeneous variances. The data were also screened for outlying values. There was strong positive correlation (.78) between the alertness task conditions; hence in order to reduce the number of dependent variables in the regression analysis, these measures were combined by standardizing the values and pooling them.

The assumption of a linear relationship between the dependent variable and predictors was checked by plotting the data (LOWESS curves) and by a lack of fit test. In order to ascertain the linearity between the dependent variable and predictors, many of the predictors were transformed into dichotomous ordinal variables. Buprenorphine doses up to 16 mg were considered as low dose and higher values as high. This was done because the dose-dependence of buprenorphine pharmacodynamics is not linear [33]. BZD doses were considered as low if lower than 20 mg and higher if 20 mg or above. The number of prescribed psychoactive drugs, other than OST or BZD drug, was considered as low up to one drug, and high if two or more other drugs. Duration of OST was considered as short if between six and twelve months, and long if above this. Substance abuse in the previous month was dichotomized as high vs. low frequency of abuse. Abstinence or substance abuse up to two days a week was considered as low-frequency substance abuse, and values above this as high-frequency substance abuse. This was based on findings showing that a mean three days of substance abuse a week is associated with a worsening of psychosocial and cognitive problems [35-37]. Substance abuse age of onset was considered as early onset up to 14 years of age, and as late onset age if 15 years of age or higher. This was based on findings showing that substance abuse onset before 15 years is especially hazardous to psychosocial and cognitive development [38]. Education was considered as low if no other than primary education had been completed, and as high if any secondary education had been completed. Homogeneity of error variance (homoscedasticity) was confirmed graphically by plotting the standardized residual against the predicted values. Independence of errors was checked using the Durbin-Watson test. Normality of residuals was checked by normality plots and using the Shapiro-Wilk’s test. Because our main interest was to examine drug treatment variables as predictors of cognitive performance, we employed multiple sequential/hierarchical linear regression analysis. First, the full model was examined as follows. Demographic variables, substance abuse variables, and the number of tests (one vs. more than one), were first entered into the model as control variables. Demographic variables included sex, level of education, and age if the test values were not age-corrected initially. Substance abuse variables included age of onset of substance abuse and frequency of substance abuse in the past month. Control variables were retained in the subsequent reduced model only if they gave a statistically significant (*p* < 0.05) contribution to the full model as a block or individually. The number of tests was also checked for the direction of association, with a positive association indicating a practice effect of repeated testing. Drug treatment variables included opioid drug type (buprenorphine vs. methadone), BZD treatment (yes vs. no), the number of psychoactive drugs (other than opioid or BZD drugs), and duration of OST. All drug treatment variables were entered sequentially into the reduced model. Unless otherwise stated, explained variance (R^2^) is reported as an adjusted value, and the regression coefficient as a standardized value (beta). All statistical analyses were done by SPSS statistical software, version 20.0, with the exception of effect size calculations, which were done by an effect size calculator devised by Durham University, UK [39]. Effect size estimations were corrected by Hedge’s correction for sample size bias.

### Cognitive tests

**Attention** was assessed by two tasks from the Test for Attentional Performance (TAP) [40]. In the Alertness task, the participant is instructed to respond to visual stimuli by pressing a response key as quickly as possible. The stimuli are presented without and with an auditory warning signal. The ‘without’ condition is a simple reaction time task reflecting tonic alertness. The ‘with-auditory-warning-signal’ condition reflects both tonic and phasic alertness. Age corrected values were used in analyzing reaction time results.

**Working memory** was assessed by the Letter-Number Sequencing task from the Wechsler Memory Scale-III. In this test the participant is instructed to repeat letters and numbers in specific order [41]. Age corrected values were used.

**Verbal memory** was assessed by the Logical Memory from the Wechsler Memory Scale-III [41]. However, only immediate recall was tested and one story used. For those participants tested repeatedly a different story was given than previously. **Visual memory** was assessed by the Benton Visual Retention Test [42].

## Results

### Group comparisons

Buprenorphine-treated patients showed statistically significantly faster simple reaction times in comparison to methadone-treated ones (the ‘without warning signal’ condition of the alertness test; (*F*(1, 100) = 7.54, *p* = 0.028). No other significant differences emerged. (All test results shown as an Additional file 1).

### Predictors of attention performance

When control variables were first entered into the full model they could predict only 1.3% of the performance variance (2.8% in the sample) of the combined alertness measure. In contrast, drug treatment variables as a block could predict an additional 6.3% (9.7% in the sample). The increment of drug treatment variables as a block significantly improved the model (*F*(4, 93) = 2.59, *p* = 0.041), but the full model remained statistically non-significant (*p* = 0.12). None of the individual predictors turned out to be significant in the full model. When the reduced model including only the drug treatment variables was tested, the OST drug group turned out to be the only significant predictor in the model (beta = .20, *t*(97) = 2.09, *p* = 0.040). The reduced model was significant (R^2^(adjusted) = .056, *F*(4, 97) = 2.51, *p* = 0.047).

In order to examine the hypothesis that reaction times are predicted by different drug treatment variables in buprenorphine- vs. methadone-treated patients, the reduced model including drug treatment variables was used. As shown in Table 3, in the buprenorphine group, being on BZD drug treatment was the only significant predictor in the model. In the methadone group, the high number of other psychoactive drugs was the best and only significant predictor in the model. Adding methadone dose to the model made it significant, although the negative association of methadone dose was not independently significant.

**Table 3:** Hierarchical regression results for combined reaction times in the Alertness test by opioid drug group

**Buprenorphine-treated patients (n = 51)**				
**Predictors in the reduced model**	**Step1 Beta(*t*-test)**^**a,b**^	**Step2 Beta(*t*-test)**	**Step3 Beta(*t*-test)**	**Step4 Beta(*t*-test)**
Drug treatment variables				
BZD treatment (yes vs. no)	.34	.36	.37	.38
	*t*(49) = 2.51	*t*(48) = 2.64	*t*(47) = 2.60	*t*(46) = 2.60
	*p* = 0.015*	*p* = 0.011*	*p* = 0.012*	*p* = 0.013*
Buprenorphine dose (high vs. low)^c^		-.16	-.17	-.19
The number of psychoactive drugs, other than opioid or BZD (high vs. low)^d^			.05	.05
Duration of OST (long vs. short)^e^				-.05
R^2^ (adjusted)	.096	.103	.084	.069
Model (ANOVA)^a^	*F*(1,49)= 6.29	*F*(2,48)= 3.88	*F*(3,47) = 2.58	*p* = 0.12
	*p* = 0.015*	*p* = 0.027*	*p* = 0.065#	
Change (ANOVA)^a^		*p* = 0.24	*p* = 0.76	*p* = 0.71
**Methadone-treated patients (n = 51)**				
**Predictors in the reduced model**	**Step1 Beta(*t*-test)**^**a,b**^	**Step2 Beta(*t*-test)**	**Step3**^**f**^**Beta(*t*-test)**	
Drug treatment variables				
The number of psychoactive drugs, other than opioid or BZD (high vs. low)	.27 #	.30 *	*.*31 *	
	*t*(49) = 1.92	*t*(48) = 2.21	*t*(47) = 2.09	
	*p* = 0.060#	*p* = 0.032*	*p* = 0.042*	
Methadone dose BZD treatment (yes vs. no)		.26 #	.26 #	
		*t*(48) = 1.88	*t*(47) = 1.86	
		*p* = 0.066#	*p* = 0.069#	
			-.02	
R^2^ (adjusted)	.051	.098	.073	
Model (ANOVA)^a^	*F*(1,49) = 3.71	*F*(2,48) = 3.72	*F*(3,47) = 2.43	
	*p* = 0.060#	*p* = 0.032*	*p* = 0.077 #	
Change (ANOVA)^a^		0.066 #	0.90	

^a^Only *p*-value shown when *p* ≥ 0.10.

^b^Signs of beta values are reversed so that positive values refer to slowing of reaction times.

^c^Considered as low up to 16 mg.

^d^Considered as low up to one drug.

^e^Considered as short when between six and twelve months.

^f^Step 4 is not shown because ‘the duration of OST’ variable correlated strongly (.61) with methadone dose producing a multicollinearity condition, and on a theoretical basis it was excluded from the analyses.

*p < 0.05. #*p* < 0.10.

### Predictors of working memory performance

The full model including control variables predicted 8.2% of the variance (16.4% in the sample). The model as a whole was significant (*F*(8, 93) = 2.28, *p* = 0.028). None of the control variables as a block or individually gave a significant contribution to the model. Consequently the control variables predicted a very low proportion of the variance (−2.5%). In contrast, the drug treatment variables as a block significantly improved the full model (*F*(4, 93) = 4.13, *p* = 0.004) predicting 11.7% of the variance above the control variables. Therefore, the control variables were removed from the model. As shown in Table 4, treatment with a BZD drug was negatively associated with working memory performance while being more than one year in OST was positively associated with working memory performance. The BZD drug treatment effect was significant but the duration of the treatment effect only approached significance. Finally, the predictive power of the drug treatment variables including the BZD variables (type or dose) on working memory performance was tested using the group including only patients with BZD in their drug regimen (n = 75). However, this model had very low predictive power on working memory (− 0.6%) and was statistically non-significant (*p* = 0.48).

**Table 4:** Hierarchical regression results for working memory (n = 102)

**Predictors in the reduced model**	**Step 1 Beta(*t*-test)**^**a**^	**Step 2 Beta(*t*-test)**	**Step 3 Beta(*t*-test)**	**Step 4 Beta(*t*-test)**
Drug treatment variables				
BZD treatment (yes vs. no)	-.34	-.30	-.28	-.28
	*t*(100) = 3.56	*t*(99) = 3.14	. *t*(98) = 2.83	*t*(97) = 2.81
	*p* = 0.0006 ***	*p* = 0.002 **	*p* = 0.006 *	*p* = 0.006 *
Duration of OST (long vs. short)^b^		.17	.17	.16
		*t*(99) = 1.81	*t*(98) = 1.72	*t*(97) = 1.69
		*p* = 0.074 #	*p* = 0.088 #	*p* = 0.094 #
The number of psychoactive drugs, other than opioid or BZD (high vs. low)^c^			-.06	.07
OST drug type (buprenorphine vs. methadone)				-.03
R^2^ (adjusted)	.104	.124	.119	.100
Model (ANOVA)^a^	*F*(1,100) = 12.68	*F*(2,99) = 8.12	*F*(3,98) = 5.52	*F*(4,97) = 4.12
	*p* = 0.0006 ***	p =0.0006 ***	*p* = 0.002 **	*p =* 0.004 **
Change (ANOVA)^a^		*F* (1,99) = 3.26		
		*p* = 0.074#	*p* = 0.52	*p* = 0.76

^a^Only *p*-value shown when *p* ≥ 0.10.

^b^Considered as short when between six and twelve months.

^c^Considered as low up to one drug.

***p < 0.00. **p < 0.01. *p < 0.05. #*p* < 0.10.

### Predictors of memory performance

When repeated testing was entered as the first variable of the full model, it was significantly associated with verbal memory performance (beta = .36, *t* (93) = 3.49, *p* = 0.0007). Therefore, in order to eliminate the significant effect of repeated testing from the model, a model including only patients tested once was formed (n = 74). Because demographic variables had minimal effect in the initial full model (data not shown), this block was dropped from the next model. Thus, the model included substance abuse variables and drug treatment variables. Because age of onset of substance abuse (early vs. late) showed a non-significant effect in the model, it was dropped from the final model. As shown in Table 5, high-frequency substance abuse and a high number of other psychoactive drugs (other than opioid or BZD drug) were the only individual significant predictors of verbal memory performance, both of which were associated negatively with verbal memory performance.

**Table 5:** Hierarchical regression results for verbal memory (n = 74)

**Predictors in the reduced model**	**Step 1 Beta(*t*-test)**^**a**^	**Step 2 Beta(*t*-test)**	**Step 3 Beta(*t*-test)**	**Step 4 Beta(*t*-test)**	**Step 5 Beta(*t*-test)**
Substance abuse variable					
Frequency of the previous month substance abuse (high vs. low)^b^	-.35	-.34 **	-.36 **	-.35 **	-.36 **
	*t*(72) = 3.17	*t*(71) = 3.24	*t*(70) = 3.34	*t*(69) = 3.22	*t*(68) = 3.15
	*p* = 0.002**	*p* = 0.002**	*p* = 0.0013**	*p* = 0.002**	*p* = 0.002**
Drug treatment variables					
The number of psychoactive drugs, other than opioid or BZD (high vs. low)^c^		-.32	-.35	-.35	-.35 **
		*t*(71) = 3.06	*t*(70) = 3.15	*t*(69) = 3.13	*t*68) = 2.99
		*p* = 0.003**	*p* = 0.002**	*p* = 0.003**	*p* = 0.004**
BZD treatment (yes vs. no)			.10	.10	10
OST drug type (buprenorphine vs. methadone)				-.03	-.03
Duration of OST (long vs. short)^d^					.01
R^2^ (adjusted)	.110	.203	.199	.189	.177
Model (ANOVA)	*F*(1,72) = 10.02	*F*(2,71) = 10.28	*F*(3,70) = 7.06	*F*(4,69) = 5.24	*F*(5,68) = 4.14
	*p* = 0.002**	*p* = 0.0001***	*p* = 0.0003 ***	*p* =0.001***	*p* =0.002 *
Change (ANOVA)^a^		*F*(1,71) = 9.37			
		*p* = 0.003 **	*p* = 0.40	*p* = 0.79	*p* = 0.91

^a^Only *p*-value shown when *p* ≥ 0.10.

^b^Considered as high when three or more days a week. Alcohol use was taken into account if it was at least mean weekly 16 portions (12 g) for females and 24 portions for males or binge drinking occurred on any day.

^c^Considered as low up to one drug.

^d^Considered as short when between six and twelve months.

***p <0.001. **p < 0. 01. *p < 0.05. #*p* < 0.10.

The full or reduced model predicting visual memory showed only low and non-significant values for all predictors. Thus, the predictive power of the models remained non-significant (*p* = 0.33 and *p* = 0.85, respectively).

## Discussion

The aim of the study was to examine the predictive power of drug treatment variables on specific cognitive performance measures in multidrug-treated opioid-dependent patients. Also, we were interested in finding out which of the possible significant associations turn out as hypothesized. All patients had been in OST for at least six months, and there were no major changes in their drug regimen within the last week prior to the study. Being on methadone-treatment predicted a rather low, though statistically significant proportion (about 5%) of attention performance as measured by combined reaction times in alertness tests. When opioid drug groups were analyzed separately about 10% of attention performance variance could be explained in both groups, but the predictors were different. Being on BZD drug treatment predicted about 10% of working memory performance. Having more than one other psychoactive drug (than an opioid or BZD drug) was negatively associated with verbal memory performance. Also, recent high-frequency substance abuse was negatively associated with verbal memory performance. Together these factors predicted about 20% of verbal memory performance variance.

### Drug treatment variables as predictors of attention performance

In buprenorphine patients co-treatment with a BZD drug was negatively associated with attention performance, but buprenorphine dose had no significant effect. This is in line with an experimental study showing that BZD diazepam in combination with buprenorphine affects negatively on reaction time, and the effect is independent of the buprenorphine dose administered [21]. In methadone patients the interpretation is more complex. The highest predictor of slower reaction time in methadone patients was treatment with more than one other psychoactive drug than methadone or BZD (beta .31). As hypothesized, there was a positive slope between methadone dose and the combined reaction time (.26) among methadone-treated patients. It is known that rapid elevation of methadone dose reduces peripheral blood oxygen saturation even among patients highly tolerant to methadone [43]. Oxygen saturation reduction has been associated with specific reaction time deficit with relative sparing of other cognitive functions [44]. Specific to methadone it has been shown by Lintzeris et al. that when a higher than normal (150%) dose of methadone is given to methadone-treated opioid-dependent patients, reductions in oxygen saturation and reaction time can be detected [21]. In sum, our results give support to our hypotheses that among buprenorphine patients, co-treatment with a BZD drug is associated negatively with attention performance. Methadone treatment, especially when done together with other psychoactive drugs, can be negatively associated with attention performance. Of note here is the timing of the possible opioid agonist effects: the drug plasma concentration peaks between 0.5–4 h after the dose for buprenorphine and 2–6 h for methadone [31,45-47].

### Drug treatment variables as predictors of working memory performance

Our hypothesis of an advantage for buprenorphine in working performance was not supported by the analyses. Instead, treatment with a BZD drug was negatively associated with working memory performance (Table 4). When we analyzed the possible associations of BZD drug type or dose, no significant associations were found, and the model had very low predictive power. Thus, we could not link the working performance with BZD parameters. According to the meta-analysis, BZDs in general have small or medium sized negative effects on working memory functioning as measured by Cohen’s d [24]. Although diazepam and oxazepam are probably the most widely used BZDs, few studies have examined their effects on a complex working memory measure like Letter-Number Sequencing. One study found that 5 mg of diazepam did not affect performance in the complex working memory measure, the n-back task, although it reduced frontal brain activation [48]. It is known that high BZD doses have a general sedative effect and thus have the potential to affect cognitive function, but the development of tolerance may outweigh these effects [49]. Notably, we observed that more than one year in OST was positively, although weakly associated with working memory performance (beta = .16). This observation is in line with our previous observation that working memory improves in buprenorphine patients between six and twelve months into OST [22].

### Drug treatment variables as predictors of memory performance

The high number of other psychoactive drugs (than opioid and BZD) and recent high- frequency substance abuse together predicted 20% of immediate verbal memory performance variance as measured by a story recall task. There is some evidence that verbal memory is more sensitive than other cognitive domains to the negative effects of multiple psychoactive drugs. This observation, however, may be specific to elderly patients and anticholinergic drugs [50]. Even less is known about the possible memory effects of psychoactive drug burden (as measured by the number of drugs) in combination with opioid agonists. In our recent longitudinal study, both buprenorphine- and methadone-treated patients lagged behind healthy controls in verbal memory performance as measured by story recall [22]. Surprisingly, when we extended the sample in the second part of our earlier study, the difference to healthy controls disappeared. Also, the correlation between the number of psychoactive drugs and verbal memory was positive, not negative, as in the current study. In sum, although our finding of a negative verbal memory effect of more than one psychoactive drug is in concert with our hypothesis, the specificity of this finding cannot be shown with our data. The finding that recent high-frequency substance abuse predicted poor verbal memory is in accordance with studies showing that recent frequent substance abuse impairs verbal memory performance [51,52].

### Implications

The results of this study suggest that cognitive impairment in opioid-dependent patients is more common when the patient is prescribed additional psychoactive drugs. It is also likely, although not confirmed by our study, that at least long-term BZD medication independently predicts cognitive impairment among multidrug-treated opioid-dependent patients. Fortunately, during OST many patients become more open to discussion about medication side-effects. Patients may agree that slower reaction times, associated in our study with co-medication differently in buprenorphine- vs. methadone-treated patients, can be disadvantageous in vehicle driving and many sport activities. For instance there is evidence OST patients being disproportionately involved in road traffic crashes accidents [53,54]. Working memory deficits, associated in our study with BZD co-medication, are known to impair reading comprehension, learning, and reasoning [55]. BZDs interfere with affective learning which is important in therapy [56]. Thus, combining BZD medication with psychological treatment may actually be detrimental to the long-term outcome of the treatment. The finding that one fifth of story recall performance variance was explained by two variables (high number of psychoactive drugs other than opioid or BZD drug; and high-frequency substance abuse in the previous month) is also relevant information. The optimal functioning of verbal memory is a useful resource in everyday life, work, or education [57]. All these facts may give the patient and prescriber a good reason to consider non-pharmacological treatment choices in place of polypharmacy. In some cases a realistic choice, for the time being, is to change cognitively harmful drugs like BZDs and tricyclic antidepressants for ones that are less harmful to cognition [58,59].

### Limitations

The distribution of the drug treatment variables, except opioid drug groups, was unplanned, and turned out to be highly skewed for some variables. As dichotomizations were used for these variables this reduces the statistical power of the analyses [60]. Therefore our findings about drug treatment effects are preliminary. Also, data about opioid and BZD doses may not be fully accurate, because drug screens do not detect extra doses of prescription drugs. Thus, our results do not confirm a causal association between drug treatment variables and cognitive performance. Patients with a higher number of prescribed drugs may have premorbid cognitive deficits that explain the associations found. On the other hand, longitudinal studies of patient groups other than OST patients have shown that discontinuation of a BZD drug regimen is followed by slow improvement in cognitive function and quality of life but no negative effects on sleep [61-64]. Our results support the idea that this could also be possible in opioid-dependent patients as well. There has been progress in the classification of psychoactive drugs by their interaction potential with buprenorphine or methadone [5,58] Alternatively, the recently formed drug burden index could be a promising tool for reducing the number of drug treatment variables in clinical studies [65]. These tools were found, however, to be unsuitable for our purposes. Negative cognitive effects of psychoactive drugs usually diminish during long-term use, and there may be differences in this between attention and memory effects [22,59,66,67]. If we had had data on length of drug use this would have been an important variable in analysing drug treatment effects. Our regression analyses were restricted only to the main effects of variables. Perhaps interactions between the variables could have explained more of the variation in cognitive performance. This was not considered appropriate given the high number of variables. Repeated testing of a third of patients is a potential confounder in the results, although after an interval of six months between testing times, as was the case in our study, the effects of repeated testing are trivial or non-existent for most measures [68,69]. In our study the verbal memory test, the Logical Memory test, was an exception, and this was taken into account in the analyses. Inclusion of psychiatric control variables, as used by Loeber et al. or Prosser et al. when studying cognitive performance in opioid-dependent patients, would probably have raised the predictive power of the analyses [70,71], and consequently the specificity of the findings.

## Conclusions

While the causal direction of effects cannot be assured with these data, the results agree with the idea that specific prescription drug treatment variables may predict poor cognitive performance in OST patients. Improvement in quality of life, successful psychological treatment, and work or education participation are common goals in OST programs, each of which are associated with good cognitive functioning. Our results suggest that psychoactive polypharmacy may be contradictory to these goals. This should give treatment practitioners and policy makers one more reason to monitor the rationality of polypharmacy in OST.

## Competing interests

Pekka Rapeli has given a paid lecture in training organized by Schering-Plough, the former manufacturer of buprenorphine.

## Authors’ contributions

PR planned and performed the cognitive testing and statistical analyses. He wrote the first version of the manuscript and prepared the final manuscript. HA conceived the idea of the study and advised in manuscript preparation. HK participated in the design of the study and in manuscript preparation. CF carried out psychiatric investigations. All authors have read and accepted the final manuscript.

## Supplementary Material

Additional file 1 Group comparisons of cognitive performances. Click here for file
