# Supplementary material for: Do drug treatment variables predict cognitive performance in multidrug-treated opioid-dependent patients? A regression analysis study
Source: Subst Abuse Treat Prev Policy. 2012 Nov 2;7:45. doi: 10.1186/1747-597X-7-45 (PMC3551729; doi:10.1186/1747-597X-7-45)
Supplement: Additional file 1 — Group comparisons of cognitive performances. [file 1747-597X-7-45-S1.docx]

# Additional files

### Group comparisons of cognitive performances

|  | Buprenorpine  (n = 104) | Methadone  (n = 104) | Group comparison (ANOVA)  *p*-value, effect size, and confidence interval^a^ |
| --- | --- | --- | --- |
| TAP Tonic Alertness/simple reaction time (ms)^b^ | 247 ± 21 | 260 ± 30 | *F*(1,100) = 5.00, *p* = 0.028*  d = 0.44 (CI95 % 0.05 – 0.83)^c^ |
| TAP Phasic Alertness/ reaction time with warning signal (ms)^b^ | 241 ± 20 | 249 ± 26 | *p* = 0.14 |
| TAP, standardized combined alertness test performance^a^ | 0.181± 0.82^c^ | - 0.181± 1.03 | *F*(1, 100) = 3.87 *p* = 0.052  d = 0.39 (CI95 % -0.01 – 0.78) |
| The Letter-Number Sequencing, standard score^b^ | 8.4 ± 2.8 | 8.7 ± 2.9 | *p* = 0.72 |
| Logical memory, immediate | 13.4 ± 3.8 | 13.3 ± 4.2 | *p* = 0.91 |
| Benton Visual Retention Test^d^ | 7.4 ± 1.4 | 7.5 ± 1.5 | *p* = 0.77 |

^a^Only *p*-value shown when *p* ≥ 0.10.

^b^n = 102.

^c^Statistics were done after subjecting the values to reciprocal transformations.

^c^Higher number indicates better performance.

^d^n = 98.

**p* < 0.05, #*p* < 0.10.

…
